# The Common Task

**Published:** 1907-05-25

**Authors:** 


### THE COMMON TASK.

Correspondence and Queries for this section should be sent to the Editor of The Hospital, 28 Southampton Street, Strand, London, and marked "Nursing Administration."

### Ward Duty and the Private Staff.

Is it desirable to use the private staff attached to a general hospital as a reserve for special cases and for filling the vacancies caused by nurses on leave? This is a question which meets with very different replies. In some London hospitals the private staff is worked in close connection with that of the hospital, and the frequent return to the wards of the nurses sent out by the hospital, whereby they are kept in touch with the newest methods, is advertised as a distinct advantage to the public. The nurses as a rule regard it as a favour, and there is so much to stimulate and interest in the wards that a skilled nurse will even take up probationer's work for a short period in order to return to the atmosphere she loves. It is very different, however, in provincial hospitals. There the introduction of members of the private staff into the wards is seldom an unmixed boon. It may be good for the nurse, but as she is generally unaware of this fact, and somewhat reluctant to return to a routine and discipline from which she considers herself emancipated, she is apt to prove a very unwelcome feature from the point of view of the ward sister. The tone of the ward suffers, and even the patients are conscious that the temporary nurse finds them uninteresting, and has no particular motive for doing her best. In one provincial hospital, these drawbacks are being felt so strongly that an effort is in progress to sever the private staff definitely from any connection with the ward work, and this although the general tone of the training school, as well as of the private staff, is exceptionally high. The abiding difficulty in provincial hospitals is the over-importance of little things, and it is exactly in this direction that the return of the former probationer to her old ward is apt to tell injuriously. Instead of being absorbed into what Henry James, on the brink of a mighty river, happily described as "the large indifferent ease in its pace and motion as of some great benevolent institution smoothly working," the private nurse is altogether too prominent and self-important, even with the best intentions, when she picks up old duties, unmodified by the smallest variation in theory or practice. It would be interesting to know the general experience of superintendents of nurses in this connection.

### The Hospital Garden.

The dearth of profitable gardening in hospitals has always been a source of surprise to those who know how very easily a patch of ground may be made to bear a rich return. While large sums are spent over and above what is necessary year by year on meat, the, amount spent on fruit and vegetables is curiously inadequate in many institutions for the wellbeing of the inmates. It matters little that patients may be provided with no vegetable but potato, since their stay is often confined to three weeks or a month, during part of which they are probably on very restricted diet. But for the health of the workers a varied supply of vegetables and fruit is absolutely essential, and we have been astonished to look through the accounts of hospitals, liberally victualled in other respects, and note the parsimonious dimensions of the greengrocer's bill. What can be done in hospital gardening is shown by the Royal Hants County Hospital at Winchester. The garden is charmingly situated, sloping away from the main building, and flanked by spacious lawns where the patients can sit. It is well stocked with fruit trees and a plentiful supply of gooseberries, currants, strawberries, etc., is available for nurses and patients, with sufficient surplus to make jam for the whole household for the year. Of ordinary vegetables there is no stint, and in due season the staff and patients alike are regaled with green peas, beans, and other garden delicacies. One gardener and a boy suffice to deal with the vegetable garden and the hospital grounds, and a good variety is maintained all the year round. The excellent plan is adopted of buying in the garden stuff at market prices and reckoning this off against the cost of labour, seeds, manure, etc. In this way all the profit made on the garden is brought to light, and, what is even more important, the exact cost of provisions is ascertained. Naturally, as the supplies are home-grown, the vegetable and fruit account is comparatively high, the hospital being able to afford liberal allowances of their own produce. In London, where fruit and vegetables are cheaper than in any part of England, it ought to be possible to provide continual variety of vegetables for the staff, and fresh fruit ought not to be absolutely banished from the dietary. An apple apiece for breakfast every morning would prove a great help in keeping a big household in health, and it is a little luxury which can be obtained at very small cost, throughout the greater part of the year.

### Robert Burns' Nurse.

A pretty compliment was paid to some district nurses in Scotland the other day on the ground of their connection with the birthplace of Jessie Lewars, to whom Burns wrote one of his happiest lyrics. His lines to her on her recovery from a dangerous illness are less well known:—

> But rarely seen since Nature's birth
> The natives of the sky;
> Yet still one seraph's left on earth,
> For Jessie did not die.

He greeted her with these lines on her first appearance after her illness, saying, with a smile:—"I knew you would get better; you have much to do before you die, believe me." And the work she had to do, the work through which her memory lives, was to nurse him in his own last illness, and close his eyes in death.

